# Prevalence of Vitamin D Deficiency and Its Association With PTH and Type 1 Diabetes Mellitus (T1DM) in a Multicultural Pediatric Population in the United Arab Emirates, 2023

**DOI:** 10.1111/1753-0407.70219

**Published:** 2026-09-15

**Authors:** Mariam K. Ibrahim, Sara Khan, Karim Siam, Shiefa Sequeria, Rubina Lone

**Affiliations:** ^1^ College of Medicine, Mohammed Bin Rashid University of Medicine and Health Sciences, Dubai Health Dubai UAE; ^2^ Pediatrics, Dubai Health Dubai UAE

**Keywords:** bone health, glycemic control, parathyroid hormone, Type 1 Diabetes mellitus, United Arab Emirates, vitamin D

## Abstract

**Aims/Introduction:**

The vitamin D and parathyroid pathways are intrinsically linked to the regulation of calcium levels within a physiological range. Type 1 Diabetic patients are in turn theoretically impacted by these factors due to insulin's dependence on calcium for its function. This paper investigates the relationship between Type 1 Diabetes Mellitus, vitamin D deficiency, and parathyroid hormone levels in the pediatric population.

**Materials and Methods:**

This is a cross‐sectional study with 102 participants aged 3–18 years old who were selected from Salama and Cerner patient databases at Al Jalila Children's Specialty Hospital (Dubai Health). Inclusion criteria for selection were based on participants' Type 1 Diabetes Mellitus status and Hemoglobin A1c levels. Furthermore, other factors such as phosphate levels, calcium levels, and comorbidities were collected.

**Results:**

The prevalence of vitamin D insufficiency was 73.3%, with 63.2% of patients reporting normal parathyroid hormone levels. There was no significant correlation between vitamin D and parathyroid hormone levels (*χ* = 7.7, *p* = 0.103). Of the diabetic patients with insufficient vitamin D levels and normal parathyroid hormone levels, 83.3% had normal calcium levels (*χ* = 22.986, *p* = 0.028). There was no significant correlation between vitamin D, parathyroid hormone, and other factors such as phosphate levels, vitamin D supplementation, and comorbidities.

**Conclusion:**

Type 1 Diabetes pediatric patients exhibited the ability to regulate calcium homeostasis through parathyroid hormone regulation despite having insufficient vitamin D levels. However, such compensatory mechanisms may manifest as long‐term complications without early intervention. Further investigations should be conducted to investigate the causal pathways.

## Highlights

This study shows a significant prevalence of vitamin D insufficiency with normal parathyroid hormone and calcium levels among pediatric Type 1 Diabetes Mellitus patients in the United Arab Emirates. The study promotes the need to revise global reference ranges to tailor to demographic variations:

## Graphical abstract

This cross‐sectional study conducted in the United Arab Emirates investigated Vitamin D status in 102 pediatric patients (ages 3–18) with Type 1 Diabetes Mellitus in the United Arab Emirates, revealing a high prevalence of Vitamin D insufficiency that frequently presents alongside normal parathyroid hormone levels. These findings suggest the existence of physiological compensatory mechanisms in this demographic and indicate that standard Vitamin D reference ranges may require population‐specific adjustments.

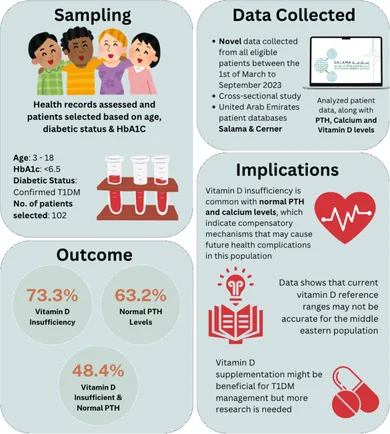

## 1 Introduction

Type 1 Diabetes Mellitus (T1DM) is a chronic autoimmune condition that is generally diagnosed in childhood and early adulthood. It is characterized by autoimmune destruction of pancreatic beta cells, leading to insulin deficiency and consequent hyperglycemia if left uncontrolled by exogenous insulin [1]. The etiological agents leading to the pathogenesis of T1DM remain elusive, but numerous studies demonstrate a complex interplay between genetic predispositions and environmental factors [1, 2, 3, 4]. T1DM has been extensively studied for a plethora of complications ranging from microvascular to macrovascular, and even bone‐related complications [5]. Unfortunately, the global incidence of T1DM is on an annual 3%–4% incline, amplifying the burden of the already highly prevalent disease affecting 8.4 million people as of 2021 [6, 7]. In the United Arab Emirates (UAE) alone, 1.4 million people were estimated to have diabetes mellitus as of 2021, of which 0.4% are adolescents with T1DM [8, 9].

Vitamin D plays a major role in human physiology, most notably for the purposes of this study: calcium homeostasis and immune function [10]. The active form of vitamin D, 1,25‐dihydroxyvitamin D [1,25(OH)2D], plays an important role in the regulation of calcium levels and consequently bone health. Vitamin D is activated through three major pathways: UV radiation of cutaneous vitamin D stores, dietary supplementation, or parathyroid hormone (PTH) in response to low circulating calcium levels [11]. Vitamin D deficiency has been considered a severe public health burden in the UAE and has mostly been explicated by changes in seasonality, sun aversion habits such as daily attire, and lack of vitamin D supplementation [12, 13, 14]. Additionally, studies have shown that vitamin D deficiency in the UAE is more common in females and UAE nationals than males and other ethnicities, which can be attributed to cultural norms [3, 5, 11]. Alarmingly, the high prevalence of vitamin D deficiency in adolescents and children raises concerns on a developmental level, as vitamin D plays a crucial role in bone development [15].

PTH is a major player in calcium homeostasis, and is the upstream regulator of renal vitamin D activation and all its downstream effects [16]. PTH also regulates several other pathways such as osteoclastic activation through the OPG/RANK/RANKL pathway leading to bone resorption, increased renal calcium reabsorption, and augmented intestinal calcium reabsorption [17, 18]. Vitamin D deficiency would lead to a decreased amount of activated vitamin D, and consequently PTH must maintain homeostasis by upregulation of the other functional pathways; this leads to the extensively studied long‐term effects, most notably bone‐related disorders caused by the decreased bone density such as osteomalacia or rickets. Intriguingly, studies now show that receptor activator of nuclear factor kappa beta ligand (RANKL) and osteoprotegerin (OPG) could function as an integrative biomarker for a range of endocrine signaling disturbances observed in T1DM patients due to their strong association with PTH, active vitamin D, and T1DM [15]. Elevated PTH levels have also been reported in patients with T1DM; this has been linked to insulin resistance and beta cell destruction [19, 20].

Essentially, calcium homeostasis and effectively circulating calcium levels are the core of the association between PTH, vitamin D, and T1DM. T1DM, along with its associated hyperglycemia, advanced glycation end‐products (AGEs), and microvascular damage, interferes with calcium regulation by impairing function and damaging calcium‐regulating organs; namely, kidneys, bones, and the small intestines, in addition to dysregulation of calciotropic hormones such as PTH [21].

Vitamin D is being extensively studied for both its protective effects against T1DM pathogenesis at sufficient levels, and its potential role in pathogenesis of T1DM at deficient levels, either due to low vitamin D intake or vitamin D receptor (VDR) polymorphism affecting vitamin D absorption [21]. Vitamin D has been shown to reduce expression of inflammatory markers associated with diabetic nephropathy, and also play a protective role in prevention of pancreatic islet cell death [10, 22]. Increased adipose tissue in patients with higher BMI, which is prevalent in T1DM patients, promotes lower circulating levels and sequestration of vitamin D [23]. Interestingly, while vitamin D deficiency is shown to play a role in T1DM pathogenesis, T1DM itself can lead to vitamin D deficiency. One possible mechanism for vitamin D deficiency in T1DM has been shown through the increased loss of vitamin D binding protein in urine [24], another is downregulation of nuclear VDR expression as a result of accumulation of AGEs [25]. Vitamin D deficiency has also been associated with increased insulin requirements due to higher insulin resistance in both diabetic and non‐diabetic populations [26, 27, 28, 29]. Vitamin D is thought to modulate insulin secretion and sensitivity through multiple mechanisms, including calcium homeostasis regulation, insulin signaling and immunomodulation [30, 31]. Studies have also shown that there is an association between 25(OH)D, the serum form of vitamin D, and Hemoglobin A1c (HbA1c), high levels of which are a known marker for diabetes [32]. Vitamin D deficiency has been shown in several observational studies to correlate with worse glycemic control in T1DM patients, as shown by higher HbA1c values and increased insulin requirements [33, 34]. Despite these associations, studies into a definitive causal pathway linking vitamin D, PTH and T1DM are sparse. A contributing factor to the scarcity of interest in studying this association is the general lack of data to show the combined burden of T1DM and alterations in vitamin D and PTH levels. This study aims to provide novel data that has not been previously available in the UAE or the wider region to our knowledge at this point.

25(OH)D, the serum form of vitamin D, and Hemoglobin A1c (HbA1c), high levels of which are a known marker for diabetes [35]. Despite these associations, studies into a definitive causal pathway linking vitamin D, PTH, and T1DM are sparse. A contributing factor to the scarcity of interest in studying this association is the general lack of data to show the combined burden of T1DM and alterations in vitamin D and PTH levels. This study aims to provide novel data that has not been previously available in the UAE or the wider region to our knowledge at this point.

## 2 Materials and Methods

### 2.1 Study Design

This is a cross‐sectional study conducted at Mohammed Bin Rashid University of Medicine and Health Sciences (MBRU—Dubai Health) in collaboration with Al Jalila Children's Specialty Hospital (AJCH—Dubai Health), Dubai, UAE. An estimated maximum sample of 200 eligible patients was recruited through AJCH—Dubai Health's Salama and Cerner patient databases between March 1st and September 30th, 2023. Pediatric patients between the ages of 3–18 years old were selected through inclusion criteria based on their T1DM status as specified by the World Health Organization's (WHO) criteria, which indicates that a clinically confirmed diabetic patient has an HbA1c level above 48 mmol/mol (6.5%) [36].

### 2.2 Variables

The paper focuses on the following variables: age, sex, BMI, nationality, duration of diagnosis, vitamin D levels, PTH levels, comorbidities (such as co‐existing thyroid disorders, bone disorders, etc.), insulin prescription, vitamin D supplementation, serum calcium and phosphate levels. Vitamin D levels were measured through serum 25(OH)D levels and were sub‐categorized into deficient (less than 10 ng/mL), insufficient (10–30 ng/mL), and sufficient (30–100 ng/mL). PTH was sub‐categorized into low (less than 10 pg/mL), normal (10–65 pg/mL), and high (greater than 65 pg/mL). Serum calcium levels were sub‐categorized into low (less than 8.4 mg/dL), normal (8.4–10.2 mg/dL), and high (greater than 10.2 mg/dL). Serum phosphate levels were first sub‐categorized by age groups (“3‐5 years old,” “6‐13 years old,” “14‐16 years old,” “17–18 years old”), and then classified into low, normal and high based on each age group and each sex, as reported according to AJCH—Dubai Health standards. Vitamin D supplementation and insulin prescription were categorized as binary “yes/no” variables. Data on patients' coexisting comorbidities was also collected as a binary “yes/no” variable. Such disorders included thyroid disorders (e.g., Graves Disease, Hashimoto's Disease, etc.), bone disorders (e.g., growth hormone deficiency) and genetic disorders (e.g., G6PD deficiency).

### 2.3 Data Sources/Measurement

Blood samples collected under the routine metabolic blood panel ordered for diabetic patients were retroactively and independently analyzed through the laboratories at AJCH—Dubai Health. Glycated HbA1c levels were measured using the Alinity c Hemoglobin A1c Reagent Kit. Vitamin D levels were measured using the Alinity i 25‐OH Vitamin D Reagent Kit. PTH levels were measured using the Alinity i Intact PTH Reagent Kit. Data were then extracted from Salama and Cerner patient databases. All de‐identified data were stored on password‐encrypted files on password‐protected computers.

### 2.4 Bias

Given that the data was collected from a single center, there is inherent selection bias. The study could also not control for vitamin D level confounders such as seasonality and diet due to feasibility and lack of corresponding data in patient files. Studies have shown that season and dietary intake play a role in vitamin D levels [10, 37, 38].

### 2.5 Quantitative Variables

The study investigated a wide range of quantitative variables, but of considerable significance were BMI, duration of diagnosis, and vitamin D, PTH, calcium, and phosphate levels. Vitamin D was sub‐categorized into “deficient,” “insufficient,” and “sufficient.” BMI was sub‐categorized into “underweight,” “healthy weight,” “overweight,” and “obese” as defined by the Centers for Disease Control and Prevention (CDC) [39]. Duration of diagnosis was sub‐categorized as “0–1 years,” “2–5 years,” and “6+ years.” PTH, calcium, and phosphate were also sub‐categorized into “low,” “normal,” and “high.” The patients were then grouped into nine categories based on their vitamin D and PTH levels (shown in Table 1). This was done to enable analysis of these groups with the other variables such as calcium levels, phosphate levels, and comorbidities.

**TABLE 1:** Association between insulin intake, BMI, calcium and HbA1c by vitamin D categories.

		Vitamin D categories						*p*
		Deficient		Insufficient		Sufficient		
		Count	%	Count	%	Count	%	
Insulin	No	0	0.0%	18	81.8%	4	18.2%	0.949
	Yes	5	7.8%	45	70.3%	14	21.9%	
Calcium	Low calcium	2	50.0%	2	50.0%	0	0.0%	0.655
	Normal calcium	1	2.2%	37	80.4%	8	17.4%	
	High calcium	1	9.1%	8	72.7%	2	18.2%	
HbA1c_R^a^	Normal ≤ 6.5	0	0.0%	0	0.0%	3	100.0%	0.006
	Poor > 6.5	5	6.0%	63	75.9%	15	18.1%	

*Note:* Chi‐square tests were done by combining vitamin D category deficient with insufficient.

^a^ Statistically significant (*p* < 0.05).

### 2.6 Statistical Methods

Statistical Package for Social Sciences (SPSS), version 28.0 was used to analyze the data. The statistical significance was fixed at the level of 5%. Frequency tables were generated for all categorical variables. A non‐parametric comparison test, Pearson's chi‐square test, was used to determine the significance of the pre‐defined nine categories of vitamin D and PTH levels among T1DM patients. The chi‐square test was also conducted to determine the significance of the pre‐defined nine categories correlated with vitamin D supplementation, calcium levels, phosphate levels, and comorbidities. Missing data for any variable studied was excluded from analysis by presenting the data in valid percentages.

## 3 Results

### 3.1 Descriptive Data

Within the selected time frame, out of the 200 patients sampled at AJCH—Dubai Health, 102 patients fell into the pre‐defined inclusion criteria. Of the 102 patients selected, 52.9% were Emirati and 47.1% were non‐Emirati. The non‐Emirati nationalities included “Egyptian,” “Pakistani,” “Indian,” “Canadian,” “Filipino,” etc. 70.6% of patients were aged 6–13 years. 55.9% of patients were female. 61.5% were underweight, 12.1% were overweight, and 2.2% were obese. For the duration of diagnosis, 60.6% were diagnosed in the last year (0–1 years) (see Table 2).

**TABLE 2:** Demographic data of Type 1 Diabetic pediatric patients (*n* = 102).

Characteristics	*n*	%
Age		
3–5	16	15.7
6–13	72	70.6
14–16	12	11.8
17–18	2	2.0
Missing	0	—
Sex		
Male	45	44.1
Female	57	55.9
Missing	0	—
BMI^a^		
Underweight	56	61.5
Healthy	22	24.2
Overweight	11	12.1
Obese	2	2.2
Missing	11	—
Nationality		
Emirati	54	52.9
Non‐Emirati	48	47.1
Missing	0	—
Duration of diagnosis		
0–1 years	60	60.6
2–5 years	24	24.2
6+ years	15	15.2
Missing	3	—

*Note:* Valid percent reported.

^a^ BMI ranges: underweight ≤ 18.5 kg/m^2^, healthy = 18.5–24.9 kg/m^2^, overweight = 25.0–29.9 kg/m^2^, obese ≥ 30 kg/m^2^.

### 3.2 Outcome Data

The prevalence of categorized vitamin D levels and PTH levels were collected (Table 3). Of the 102 patients, 5.8% were vitamin D deficient and 73.3% were vitamin D insufficient. 63.2% of the patients recorded normal PTH levels. 72.9% of the patients recorded normal calcium levels. 50.0% of patients were prescribed vitamin D supplements (the most common of which was Cholecalciferol 100 mcg). Co‐existing comorbidities were also reported, where 26.5% of patients had comorbidities. Phosphate levels were sub‐categorized based on their age and sex, with 63.3% of patients having normal phosphate levels. 69.6% of patients were prescribed insulin (e.g., Lispro, Tresiba, etc.).

**TABLE 3:** Data on clinical subcategorized biomarkers in T1DM pediatric patients (*n* = 102).

Characteristics	*n*	%
Vitamin D levels^a^		
Deficient	5	5.8
Insufficient	63	73.3
Sufficient	18	20.9
Missing	16	—
Parathyroid hormone levels^b^		
Low	13	17.1
Normal	48	63.2
High	15	19.7
Missing	26	—
Vitamin D supplements		
No supplements	51	50.0
Taking supplements	51	50.0
Missing	0	—
Calcium levels^c^		
Low	4	5.5
Normal	53	72.9
High	16	21.9
Missing	29	—
Phosphate levels^d^		
Low	17	34.7
Normal	31	63.3
High	1	2.0
Missing	53	—
Co‐existing comorbidity		
Yes	27	26.5
No	75	73.5
Missing	0	—
Insulin prescription		
Yes	71	69.6
No	31	30.4
Missing	0	—

*Note:* Valid percent reported.

^a^ Vitamin D Ranges: Deficient ≤ 10 ng/mL, insufficient = 10–30 ng/mL, and sufficient = 30–100 ng/mL.

^b^ Parathyroid Hormone Ranges: Low ≤ 10 pg/mL, normal = 10–65 pg/mL, and high ≥ 65 pg/mL.

^c^ Calcium Ranges: Low ≤ 8.4 mg/day, normal = 8.4–10.2 mg/dL, and high ≥ 10.2 mg/dL.

^d^ Phosphate ranges subcategorized based on age and sex as reported by Al Jalila Children's Specialty Hospital ranges.

### 3.3 Main Results

Pearson's chi‐square test was conducted to investigate the correlation between vitamin D levels and the following demographic data: age (*χ* = 4.395, *p* = 0.623), sex (*χ* = 2.010, *p* = 0.366), BMI (*χ* = 4.094, *p* = 0.664), and nationality (*χ* = 0.492, *p* = 0.782) (Table 4). The correlation between vitamin D levels and PTH was done using Pearson's chi square test (*χ* = 7.7, *p* = 0.103) (Table 5).

**TABLE 4:** Crosstabulation of vitamin D subcategories by age (*n* = 86), sex (*n* = 86), and BMI (*n* = 77).

	Vitamin D deficient	Vitamin D insufficient	Vitamin D sufficient	*p*
Age				0.623
3–5	1 (20.0%)	8 (12.7%)	5 (27.8%)	
6–13	3 (60.0%)	47 (74.6%)	11 (61.1%)	
14–16	1 (20.0%)	7 (11.1%)	1 (5.6%)	
17–18	0 (0.0%)	1 (1.6%)	1 (5.6%)	
Sex				0.366
Male	1 (20.0%)	29 (46.0%)	10 (55.6%)	
Female	4 (80.0%)	34 (54.0%)	8 (44.4%)	
BMI^a^				0.664
Underweight	1 (33.3%)	35 (62.5%)	14 (77.8%)	
Healthy	1 (33.3%)	13 (23.2%)	3 (16.7%)	
Overweight	1 (33.3%)	6 (10.7%)	1 (5.6%)	
Obese	0 (0.0%)	2 (3.6%)	0 (0.0%)	
Nationality				0.782
Emirati	3 (60.0%)	33 (52.4%)	11 (61.1%)	
Non‐Emirati	2 (40.0%)	30 (47.6%)	7 (38.9%)	

*Note:* Column percent reported.

^a^ BMI ranges: underweight ≤ 18.5 kg/m^2^, healthy = 18.5–24.9 kg/m^2^, overweight = 25.0–29.9 kg/m^2^, obese ≥ 30 kg/m^2^.

**TABLE 5:** Crosstabulation of vitamin D subcategories with parathyroid hormone subcategories using Pearson's chi‐square test (*n* = 64, *p* = 0.103).

		Parathyroid hormone^b^		
		Low	Normal	High
Vitamin D^a^	Deficient	0 (0%)	5 (7.8%)	0 (0%)
	Insufficient	7 (10.9%)	31 (48.4%)	6 (9.4%)
	Sufficient	4 (6.3%)	6 (9.4%)	5 (7.8%)

^a^ Vitamin D ranges: Deficient ≤ 10 ng/mL, insufficient = 10–30 ng/mL, and sufficient = 30–100 ng/mL.

^b^ Parathyroid hormone ranges: Low ≤ 10 pg/mL, normal = 10–65 pg/mL, and high ≥ 65 pg/mL.

### 3.4 Other Analyses

Subsequent mean analyses and Pearson's chi‐square tests were performed to study the correlation between vitamin D and BMI, HbA1c levels, and calcium levels (Tables 1 and 6). The vitamin D subcategories were than crosstabulated with PTH and the following variables: vitamin D supplementation (*χ* = 8.367, *p* = 0.212), serum calcium levels (*χ* = 22.986, *p* = 0.028), serum phosphate levels (*χ* = 10.862, *p* = 0.054), and co‐existing comorbidities (*χ* = 10.904, *p* = 0.091) (Table 7).

**TABLE 6:** Mean, SD of BMI, HbA1c and Ca^2+^ by vitamin D categories.

	Vitamin D categorized						*p*
	Deficient		Insufficient		Sufficient		
	Mean	SD	Mean	SD	Mean	SD	
BMI^a^	21.4	5.4	18.8	4.9	17.0	3.2	0.001
HbA1c	11.5	2.1	10.4	2.3	8.5	2.1	0.231
Ca^2+^	9.0	1.3	9.3	1.3	9.7	0.6	0.633

^a^ Statistically significant (*p* < 0.05).

**TABLE 7:** Crosstabulation of vitamin D subcategories and parathyroid hormone (PTH) subcategories with vitamin D supplementation, calcium levels, phosphate levels and co‐existing comorbidities using Pearson's chi‐square test.

	Vitamin D deficiency and low PTH	Vitamin D deficiency and normal PTH	Vitamin D deficiency and high PTH	Vitamin D insufficiency and low PTH	Vitamin D insufficiency and normal PTH	Vitamin D insufficiency and high PTH	Vitamin D sufficiency and low PTH	Vitamin D sufficiency and normal PTH	Vitamin D sufficiency and high PTH	*p*
Vitamin D supplements										0.212
Yes	0 (0.0%)	4 (80.0%)	0 (0.0%)	4 (57.1%)	14 (45.2%)	4 (66.7%)	1 (25.0%)	1 (16.7%)	4 (80.0%)	
No	0 (0.0%)	1 (20.0%)	0 (0.0%)	3 (42.9%)	17 (54.8%)	2 (33.3%)	3 (75.0%)	4 (83.3%)	1 (20.0%)	
Calcium levels^a^ ^,^ *										0.028
Low	0 (0.0%)	2 (50%)	0 (0.0%)	1 (16.7%)	0 (0.0%)	0 (0.0%)	0 (0.0%)	0 (0.0%)	0 (0.0%)	
Normal	0 (0.0%)	1 (25.0%)	0 (0.0%)	5 (83.3%)	20 (83.3%)	1 (33.3%)	2 (66.7%)	3 (100.0%)	1 (100.0%)	
High	0 (0.0%)	1 (25.0%)	0 (0.0%)	0 (0.0%)	4 (16.7%)	2 (66.7%)	1 (33.3%)	0 (0.0%)	0 (0.0%)	
Phosphate levels^b^										0.054
Low	0 (0.0%)	4 (100.0%)	0 (0.0%)	2 (50.0%)	4 (22.2%)	0 (0.0%)	1 (33.3%)	0 (0.0%)	1 (100.0%)	
Normal	0 (0.0%)	0 (0.0%)	0 (0.0%)	2 (50.0%)	14 (77.8%)	1 (100.0%)	2 (66.7%)	0 (0.0%)	0 (0.0%)	
High	0 (0.0%)	0 (0.0%)	0 (0.0%)	0 (0.0%)	0 (0.0%)	0 (0.0%)	0 (0.0%)	0 (0.0%)	0 (0.0%)	
Co‐existing comorbidities										0.091
Yes	0 (0.0%)	1 (20.0%)	0 (0.0%)	2 (28.6%)	5 (16.1%)	0 (0.0%)	2 (50.0%)	4 (66.7%)	2 (40.0%)	
No	0 (0.0%)	4 (80.0%)	0 (0.0%)	5 (71.4%)	26 (83.9%)	6 (100.0%)	2 (50.0%)	2 (33.3%)	3 (60.0%)	

*Note:* Column percent reported.

^a^ Calcium Ranges: Low ≤ 8.4 mg/day, normal = 8.4–10.2 mg/dL, and high ≥ 10.2 mg/dL.

^b^ Phosphate ranges subcategorized based on age and sex as reported by Al Jalila Children's Specialty Hospital – Dubai Health ranges.

* Statistically significant (*p* < 0.05).

## 4 Discussion

In this expansive medical study encompassing 102 patients, the acquisition of novel data has unveiled key demographic insights. The collected population exhibited a slight gender imbalance, of which 55.9% were female. Furthermore, there was a near‐even split between non‐Emirati and Emirati nationalities (47.1% vs. 52.9%). Interestingly, the cohort presented an exact 50/50 distribution concerning vitamin D supplementation on record. While a significant portion of the population (73.3%) is deficient in vitamin D, the majority exhibit normal PTH levels (63.2%) and normal calcium levels (72.9%), which indicates preserved calcium homeostasis in early pediatric T1DM.

The primary focus of the investigation, delving into the association between vitamin D levels, PTH levels, and pediatric T1DM, yielded statistically insignificant results. Consequently, further analyses on other parameters were conducted, specifically investigating the relationship of vitamin D and PTH with calcium levels, phosphate levels, vitamin D supplementation, and comorbidities. The association between the nine pre‐defined PTH‐vitamin D categories and vitamin D supplementation revealed no significant correlation. The data pointed predominantly toward a statistically significant (*p* = 0.028) prevalence of vitamin D insufficiency, accompanied by normal PTH and calcium levels, suggesting potential compensatory mechanisms at play to maintain calcium homeostasis (Table 1). This intriguing finding prompts further inquiry into the long‐term ramifications of vitamin D insufficiency on pediatric T1DM and indicates that while these mechanisms maintain calcium balance in the presence of vitamin D insufficiency, they may remain fragile and susceptible to disruption.

Vitamin D deficiency has been shown to increase insulin requirements by promoting higher insulin resistance in both diabetic and non‐diabetic populations. As highlighted earlier, vitamin D deficiency or insufficiency correlates with increased daily insulin dose and elevated HbA1c, indicating reduced insulin sensitivity and suboptimal glycemic control [33]. This aligns with the current findings: Table 1 shows a statistically significant association between vitamin D levels and HbA1c (*p* = 0.006). This strengthens the current observations in the literature showing that lower vitamin D levels correspond to an increased need for insulin dose adjustments.

Additionally, higher BMI is associated with lower vitamin D concentrations in T1DM, and both higher BMI and lower vitamin D have been linked to increased insulin requirements and poorer glycemic control [40]. Consistent with these findings, our study demonstrated a statistically significant negative correlation between BMI and vitamin D levels (*p* = 0.001, Table 6), indicating that lower vitamin D levels correspond to higher BMI. Physiologically, vitamin D has been linked to modulation of insulin sensitivity and inflammation. Several interventional and systematic reviews have shown that vitamin D supplementation reliably corrects deficiency but may also influence glycemic control and insulin dose requirements in established T1DM. Maintaining adequate vitamin D status is recommended for improved bone health and may result in metabolic benefits, including reduced insulin needs and improved glycemic stability.

Literature searches to compare the prevalence of vitamin D and PTH levels in pediatric T1DM patients were mostly limited to studies conducted in the Middle East region to account for the influence of geography and seasonality. Additionally, each study followed different reference ranges for the subcategories of the biomarkers, so an accurate comparison of prevalence was difficult to carry out.

For the demographic data, this study presented a majority of female, Emirati, and underweight patients. A study conducted in Al Ain, UAE showed that 67% of the studied population had vitamin D deficiency, of which the majority were female, Arab (including Emirati), and overweight [41]. While the current study shows a high prevalence of vitamin D insufficiency in females (54.0%) and Emiratis (52.4%), the low prevalence of overweight (10.7%) and obese (3.6%) patients could be explained by the high prevalence of newly diagnosed T1DM pediatric patients (60.6%), which was not accounted for in the Al Ain study. This is further supported by the likelihood of increased BMI with age in a T1DM diagnosis [42].

Studies have shown that vitamin D deficiency is prevalent in pediatric T1DM patients [11, 20, 43], of which one study reported different reference ranges for vitamin D levels [20]. PTH levels have mostly been reported as elevated in T1DM pediatric patients [11, 17, 44], while some studies have reported low, normal, or no correlation with T1DM [45, 46, 47, 48]. The current study showed normal PTH levels, which suggests that the suppressive regulatory mechanism in response to taking vitamin D supplements is functioning normally to counteract secondary hyperparathyroidism, as hypothesized by the study conducted by Povaliaeva et al. [45].

Due to their involvement in bone regulation, vitamin D and PTH levels were investigated in pediatric T1DM patients in correlation to fracture risk, with the results showing an association with decreased serum 25(OH)D and increased PTH [17]. Similarly, one study in Saudi Arabia found low vitamin D levels and high PTH levels in T1DM pediatric patients [39]. In comparison, a study conducted in Egypt found high levels of phosphate and PTH, and low levels of calcium and vitamin D [11]. This is consistent with this study's normal PTH and calcium levels, as it exhibits functioning regulatory mechanisms for insufficient vitamin D levels.

This study's discoveries contribute to our comprehension of the interplay among pediatric T1DM, vitamin D levels, and PTH levels, both collectively and in isolation. The alarmingly elevated prevalence of vitamin D insufficiency among children, juxtaposed with the absence of clinical manifestations, suggests a potential incongruity in the reference ranges employed to assess vitamin D levels. The existing global reference ranges, derived from a middle‐aged European male population [49], overlook variations in ethnicity and geographical location, particularly among Arab individuals in the Middle Eastern region. Moreover, evidence from other studies challenges the accuracy of these figures for diverse populations, as other studies have suggested that the required levels of vitamin D may differ depending on age, sex, and ethnicity [50]. It is crucial to revise these reference ranges to accurately assess the specific vitamin D requirements of various populations in different geographical locations. Moreover, physicians should consider vitamin D supplementation an integral part of T1DM management to maintain optimal glycemic control and to prevent further clinical manifestations stemming from the deficiency in vitamin D. While initial signs may present as fatigue and general aches, prolonged deficiency can lead to more severe complications such as rickets in children and osteomalacia in adults, both resulting from reduced bone density [51].

The major strength of this study is its provision of novel data on the prevalence of vitamin D and PTH levels in T1DM pediatric patients in Dubai and the wider region. Prior to our study, there was a paucity of data in the region which contributed to a critical lack of understanding of the implications of vitamin D deficiency and insufficiency in these patients and thus a lack of interest in studying the potential long‐term effects of those altered levels.

Another strength is the wide range of other parameters our study collected including BMI, age, sex, calcium, vitamin D supplementation, insulin prescription, phosphate, basic metabolic panel, and lipid panels. These parameters allow for a more comprehensive assessment of the burdens discussed in this paper.

The major limitation of this study is selection bias due to its single center nature. Although a major subset of AJCH—Dubai Health's admissions are referrals by nature of their specialization, the patients within our study are not representative of the wider pediatrics population in Dubai as the center's admissions are predominantly more affluent children who have received exceptional medical attention for their morbidities. Thus, this limits the generalizability of the study. This issue with internal validity unfortunately could not be avoided due to the feasibility limitations of a single center; however, future studies can look at a larger sample from multiple specialized and non‐specialized centers with a wider patient demographic to aid in extrapolating the findings to the general pediatric T1DM population.

Another limitation lies within the standardized testing panels done for T1DM patients, while those tests universally include HbA1c as a marker for diabetes, it is not standard to measure vitamin D or PTH routinely due to either a lack of insurance coverage or their current futility in the scope of treatment and management of these patients. Consequently, our study's two main parameters after patient inclusion (vitamin D and PTH) were tested retroactively on pre‐existing blood samples independently by the laboratory at Al Jalila Children's Specialty Hospital (Dubai Health). The rate at which this retroactive testing is performed limited the study's potential sample size and therefore decreased its likely generalizability.

Future longitudinal studies could aim to investigate the causal relationship between vitamin D, PTH, and T1DM. This would involve following up a cohort of pediatric T1DM patients for several years while regularly measuring certain key parameters such as vitamin D, PTH, and calcium, as well as monitoring for the occurrence of diabetes‐related complications and pathology related to altered calcium regulation. This would provide stronger evidence to support the need for vitamin D supplementation in the management of T1DM.

While some recommendations on implementing the usage of vitamin D supplementation into management guidelines exist in the literature [52, 53], longitudinal studies or randomized controlled trials could be conducted to evaluate the potential protective effects and quality of life improvements of vitamin D supplementation on health outcomes in pediatric T1DM patients in the short and long term. This would provide direct evidence that would raise calls for inclusion of vitamin D supplementation as a key part of patient management. These future studies could also aim at exploring and evaluating the optimal dosage of vitamin D supplementation for pediatric T1DM patients while controlling for the expected inherent compliance issues that arise with self‐medication and supplementation.

Longitudinal studies and genome‐wide association studies (GWAS) could follow up cohorts with known VDR gene single nucleotide polymorphisms (SNPs) which have been implicated in variations in vitamin D absorption and activity [54]. Vitamin D has also been widely implicated in the immune system, and specifically its function in building immune tolerance and thus its potential to prolong the “honeymoon phase” of T1DM [55]; therefore, a study investigating the effects of vitamin D supplementation as a protective agent against the eventual T1DM pathogenesis could highlight this function. These studies would provide information that builds an understanding of how certain genetic predeterminants could inform personalized vitamin D supplementation.

The intricate interplay between vitamin D, PTH, and T1DM plays a crucial role in regulating calcium homeostasis, maintaining bone health, and influencing glycemic control, modulating insulin sensitivity, and potentially affecting body weight in pediatric patients with T1DM. This study serves as a foundational exploration, setting the stage for future research endeavors seeking a deeper understanding of the complex dynamics between these three factors within the diverse pediatric population in the UAE. Findings demonstrate that the majority of the patients fell into vitamin D insufficient, normal PTH and normal calcium categories. The significant association between vitamin D, PTH, and calcium levels, despite the primary focus yielding insignificant results, raises questions about the compensatory mechanisms at play. Monitoring these parameters longitudinally will provide insights into the long‐term implications of vitamin D insufficiency on T1DM. Furthermore, the study emphasizes the need to revise global reference ranges, considering demographic variations, to accurately assess vitamin D requirements. Limitations include potential selection bias, restricted generalizability, constraints in routinely measuring vitamin D and PTH, and the cross‐sectional design prevents establishing causal relationships. This study represents an integral step in understanding the relationships between vitamin D, PTH, and T1DM, thus sparking a renewed interest in ultimately improving the lives of young patients suffering with this autoimmune disorder.

## Author Contributions

All listed authors: M.K.I., S.K., and K.S. had equal contributions to the conceptualization, data collection, data analysis, and manuscript drafting. M.K.I. is the research group lead, partaking in all tasks, K.S. led the manuscript and background, and S.K. did data analysis. All authors contributed equally to data collection. S.S. and R.L. both supervised the data collection, and S.S. aided in the data collection as well.

## Funding

The authors have nothing to report.

## Ethics Statement

This paper has been approved by the Institutional Review Board at MBRU—Dubai Health (MBRU‐IRB) in accordance with the procedures laid down by the University for ethical approval of all research involving human participants/samples/tissue/data.

## Conflicts of Interest

The authors declare no conflicts of interest.
